# Genome sequence of bacteriophage GiJojo, isolated using *Streptomyces mirabilis* in Catonsville, Maryland

**DOI:** 10.1128/mra.00584-24

**Published:** 2024-08-23

**Authors:** Marie Louise P. Badiola, Kaela D. Befano, McKayla L. Berger, Claudine R. Blanco, Nhyira Ghunney, Jaehyun G. Lee, Lin P. Myat, James K. Nguyen, Ellison M. Ober, Karla M. Press-Porter, Brendan T. She, Justin S. Watkins, 2023 UMBC Phage Hunters, Steven M. Caruso

**Affiliations:** 1 Department of Biological Sciences, University of Maryland Baltimore County, Baltimore, Maryland, USA; Portland State University, Portland, Oregon, USA

**Keywords:** *Streptomyces*, bacteriophages, soil microbiology, metal resistance, *Actinobacteria*, genomics, bacteriophage genetics

## Abstract

Bacteriophage GiJojo is a myovirus isolated from soil that infects *Streptomyces mirabilis* NRRL B-2400, with a genome length of 115,161 bp containing 180 genes and 29 tRNAs. Of those genes, 59 have been assigned functions. GiJojo is a member of the BS cluster of actinobacteriophages.

## ANNOUNCEMENT

Members of the *Streptomyces* genus produce antibiotics and secondary metabolites (1). *Streptomyces* bacteriophages can infect these bacteria and serve as an alternative treatment for pathogenic members of this genus (2). GiJojo is a myovirus capable of infecting *S. mirabilis* NRRL B-2400, a metal-resistant bacteria often present in polluted environments (3).

**Fig 1:**
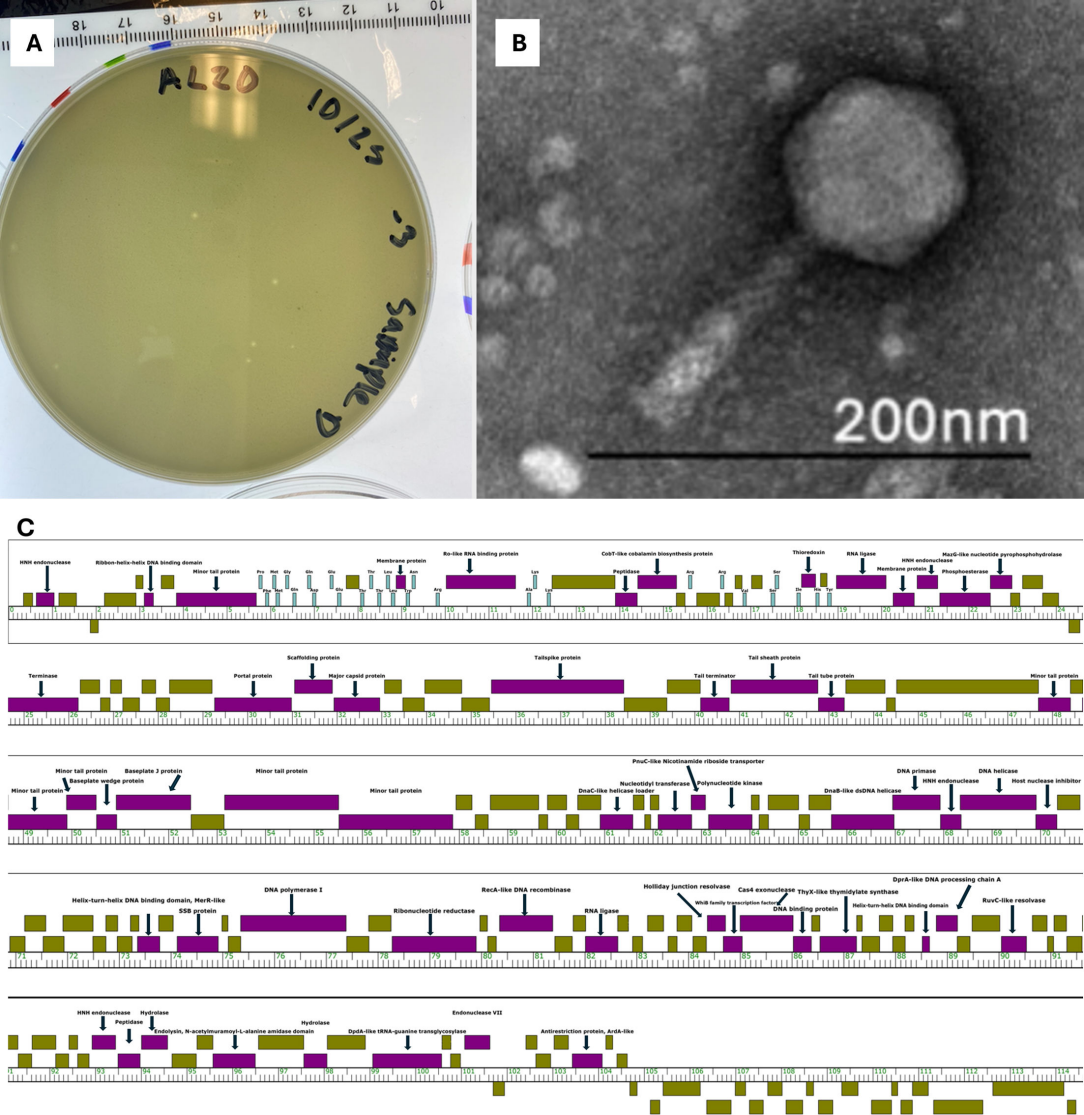
Characterization of Streptomyces myophage GiJojo**.** (**A**) Turbid, approx. 1 mm circular plaques on *S. mirabilis* infected with GiJojo incubated on nutrient agar for 24 h at 30°C. (**B**) GiJojo phage particle morphology. Fresh lysate was stained with 2% uranyl acetate on 200 mesh formvar-covered, carbon-coated copper grids, and imaged with a Morgagni M268 Transmission Electron Microscope (FEI, Hillsboro, IL, USA) at 100 kV equipped with an Orius CCD camera (Gatan Inc., Pleasanton, CA, USA). Imaging revealed bacteriophage GiJojo exhibits myoviral morphology with a contractile tail with an average tail length of 105 ± 2 nm (±SD, *n* = 4) and an average capsid diameter of 80 ± 2 nm (±SD, *n* = 4). (**C**) GiJojo’s complete genome map. GiJojo’s genome encodes 180 genes, 59 of which have assigned functions. Olive boxes denote genes with no known function, genes with putative functions are represented with the purple boxes. Twenty-nine tRNAs are represented with the teal boxes. The length of the genome and each respective gene is indicated with the ruler, with genes in the forward direction above and genes in the reverse direction below the ruler.

GiJojo was isolated from topsoil collected outside the Biological Sciences building on the campus of UMBC in Baltimore, MD, USA (39.255361°N, 76.711944°W) as part of the SEA-PHAGES program following protocols described in the *Phage Discovery Guide* (4). Briefly, the sample was mixed with phage buffer [10 mM Tris (pH 7.5), 10 mM MgSO_4_, 68 mM NaCl, and 1 mM CaCl_2_] and passed through a 0.22-µm syringe filter. The filtered sample was mixed with tryptic soy top agar (BD) containing *S. mirabilis* and poured onto nutrient agar plates (BD Difco) supplemented with 10 mM MgCl_2_, 8 mM Ca(NO_3_)_2_, and 0.5% glucose, and then incubated at 30°C for 24 h. Purification was completed by picking one isolated plaque, followed by 10-fold serial dilutions and plaque assays. This was repeated for a minimum of three rounds. Plaques demonstrated a turbid, circular, smooth-edged, and symmetrical morphology (Fig. 1A). Negative stained transmission electron microscopy of GiJojo showed myoviral morphology (Fig. 1B).

GiJojo’s DNA was isolated from fresh plate lysate using the Promega Wizard DNA Clean-Up Kit. The isolated DNA was sequenced by Illumina MiSeq utilizing the NEB Ultra II Library Kit, yielding 191,079 single-end 150 bp reads with an estimated shotgun coverage of 235×. Consed v29 was employed to verify the sequenced data assembled using Newbler v2.9 as described (5–7). Results revealed a linear double-stranded DNA chromosome with a genome length of 115,161 kbp, 53.5% GC content, and 197 bp direct terminal repeats. GiJojo was assigned to the cluster BS, which contained three additional phages in the Actinobacteriophage Database (8).

Gene annotations were completed using DNA Master v5.23.6 (9) with internal GeneMark v2.5 and Glimmer v3.02 (10, 11). GeneMark.hmm v3.25 (12) and Blastp v2.15 (13) were used to manually adjust start sites. Functions were inferred through analysis of sequence similarity determined using NCBI Blastp v2.15, structural homology assessed via HHpred v57v87 (14, 15), and synteny analysis conducted using Phamerator v561 (16). 29 tRNA encoding genes were identified and annotated using tRNAscanSE v2.0.6 and Aragorn v1.2.38 (17, 18). Of 180 identified protein-coding genes, 59 were assigned functions and 23 were identified as orphams, genes for which no homologs have been yet identified in another phage (Fig. 1C).

The conserved domain Phage_base_V (19) was found in gp88, suggesting GiJojo utilizes a secretion system homologous to the type VI secretion system used in bacterial interactions. The Phage_base_V protein domain is often found in bacterial membrane-embedded nanomachines to antagonize other prokaryotes via toxin insertion (20). This family of bacterial and phage baseplate assembly proteins forms the small spike at the end of phage or bacterial needle shafts (20).

## Data Availability

GiJojo is available in GenBank with accession no. PP725410 and Sequence Read Archive (SRA) no. SRX24123905.
